# Successful treatment of massive stage IIIB cervical cancer with a spindle cell component: A case report

**DOI:** 10.1097/MD.0000000000050727

**Published:** 2026-09-11

**Authors:** Hua Jin, Zhengri Piao, Weibo Wen

**Affiliations:** a Department of Radiation Oncology, The Affiliated Hospital of Yanbian University (Yanbian Hospital), Yanji, Jilin, China; b Department of Nuclear Medicine, The Affiliated Hospital of Yanbian University (Yanbian Hospital), Yanji, Jilin, China.

**Keywords:** bevacizumab, chemoradiotherapy, massive cervical squamous cell carcinoma, neoadjuvant chemotherapy, spindle cell component

## Abstract

**Rationale:**

Cervical cancer is one of the most common malignancies in women worldwide. Tumors larger than 10 cm in diameter are rarely encountered, and no standard protocol exists for treating them. This case serves as a reference for managing similarly giant tumors and illustrates how a sequential multimodal strategy can downstage the disease.

**Patient concerns:**

A 33-year-old woman presented with a cervical mass of 14 × 13 × 13 cm, 6 months of intermittent vaginal bleeding, and 1 month of progressive fatigue.

**Diagnoses:**

Biopsy confirmed stage IIIB cervical squamous cell carcinoma (International Federation of Gynecology and Obstetrics 2021) with a partial spindle cell component.

**Interventions:**

Because of the tumor size, neoadjuvant chemotherapy was given first to reduce tumor burden; concurrent chemoradiotherapy and bevacizumab were then administered.

**Outcomes:**

Complete remission was achieved after the planned treatment sequence. Imaging at 6 months of follow-up showed no recurrence or metastasis.

**Lessons:**

Individualized sequential multimodal therapy can produce complete remission in massive cervical squamous cell carcinoma. Neoadjuvant chemotherapy followed by concurrent chemoradiotherapy plus bevacizumab may be a curative option for selected patients with bulky stage IIIB disease.

## 1. Introduction

Cervical squamous cell carcinoma (CSCC) is one of the most common cancers in women and a leading cause of cancer death, particularly in low-resource countries.^[1,2]^

The disease most often affects women aged 35 to 55 years, and the age of onset has been decreasing in recent years.^[1]^

In advanced stages (International Federation of Gynecology and Obstetrics [FIGO] IB2–IVA), surgery is frequently unsuitable because the tumor invades the vaginal wall and pelvic tissues and may compress the ureters, causing hydronephrosis.^[3,4]^

Concurrent chemoradiotherapy (CCRT) is the standard treatment for locally advanced CSCC, but bulky tumors respond poorly and treatment-related toxicity is a major concern.^[5,6]^

Neoadjuvant chemotherapy (NACT) has been tested as a way to shrink the tumor before radiotherapy, chemotherapy, or surgery, with the aim of improving local control.^[4,7]^

Bevacizumab, a monoclonal antibody against vascular endothelial growth factor (VEGF), has also prolonged survival in advanced or recurrent cervical cancer.^[8]^

However, evidence on combining NACT, CCRT, and bevacizumab for giant CSCC lesions is scarce. We describe the diagnosis and treatment of a patient with FIGO stage IIIB CSCC presenting as a 14 × 13 × 13 cm mass who achieved complete remission (CR) after sequential NACT, CCRT, and bevacizumab. The tumor also contained a spindle cell component, an unusual histologic finding.

## 2. Case presentation

Written informed consent for publication of this case report and its images was obtained from the patient. Because this is a retrospective case report of a single patient with fully anonymized data, the Institutional Ethics Committee of the Affiliated Hospital of Yanbian University reviewed the study protocol and confirmed that formal ethics approval was not required.

A 33-year-old unmarried, nulliparous woman was admitted in June 2023. Intermittent vaginal bleeding had begun in early 2023 without an identifiable trigger and had initially gone unexamined; over the month before admission; she also developed progressive fatigue and lower abdominal pain. She had smoked approximately 15 cigarettes per day for 10 years.

**Figure 1.**
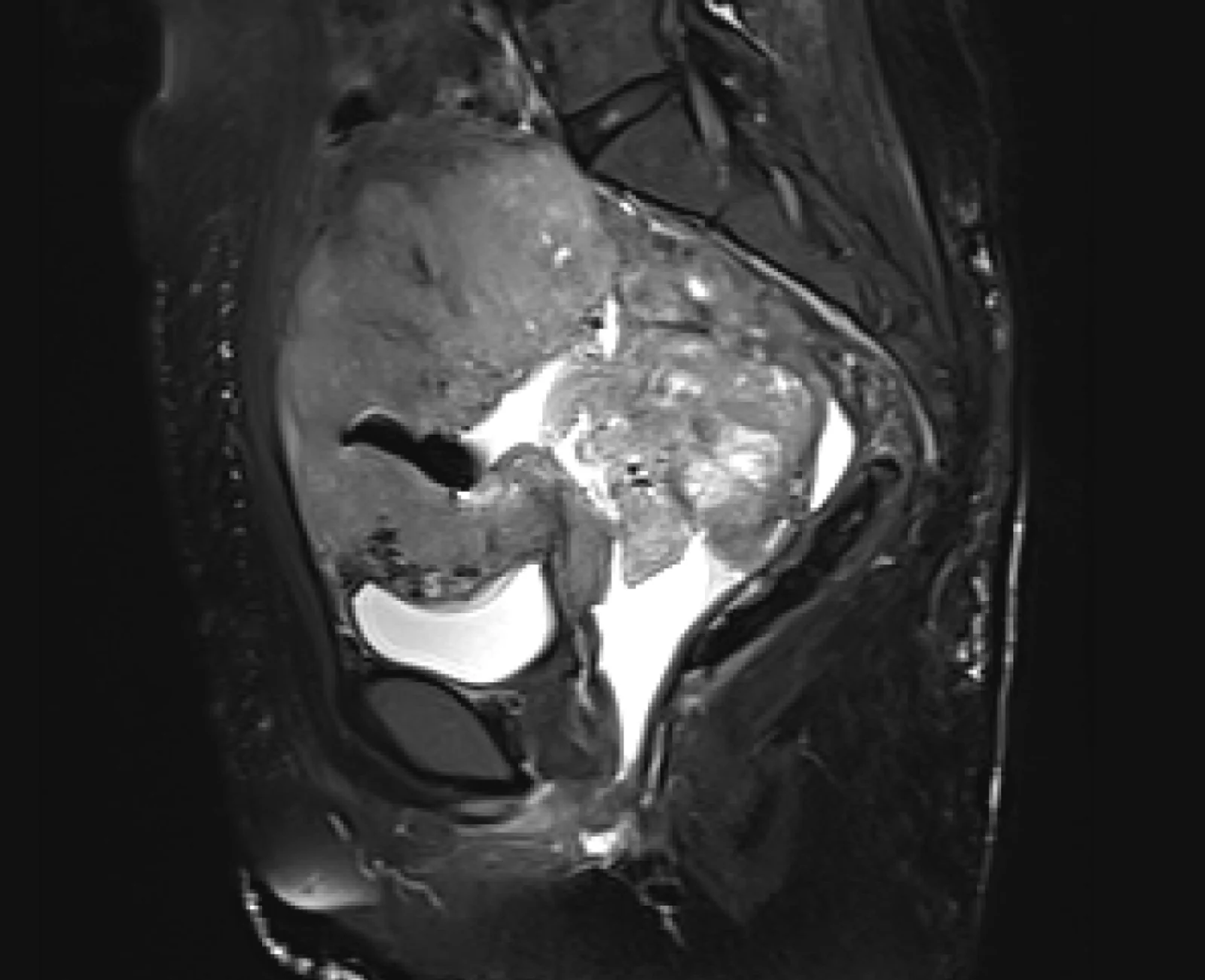
Pretreatment pelvic MRI. Sagittal T2-weighted fat-suppressed sequence acquired before NACT demonstrates a massive heterogeneous cervical mass measuring 14 × 13 × 13 cm (arrows), occupying nearly the entire pelvic cavity, with compression of the lower ureters and bilateral hydronephrosis. MRI = magnetic resonance imaging, NACT = neoadjuvant chemotherapy.

**Figure 2.**
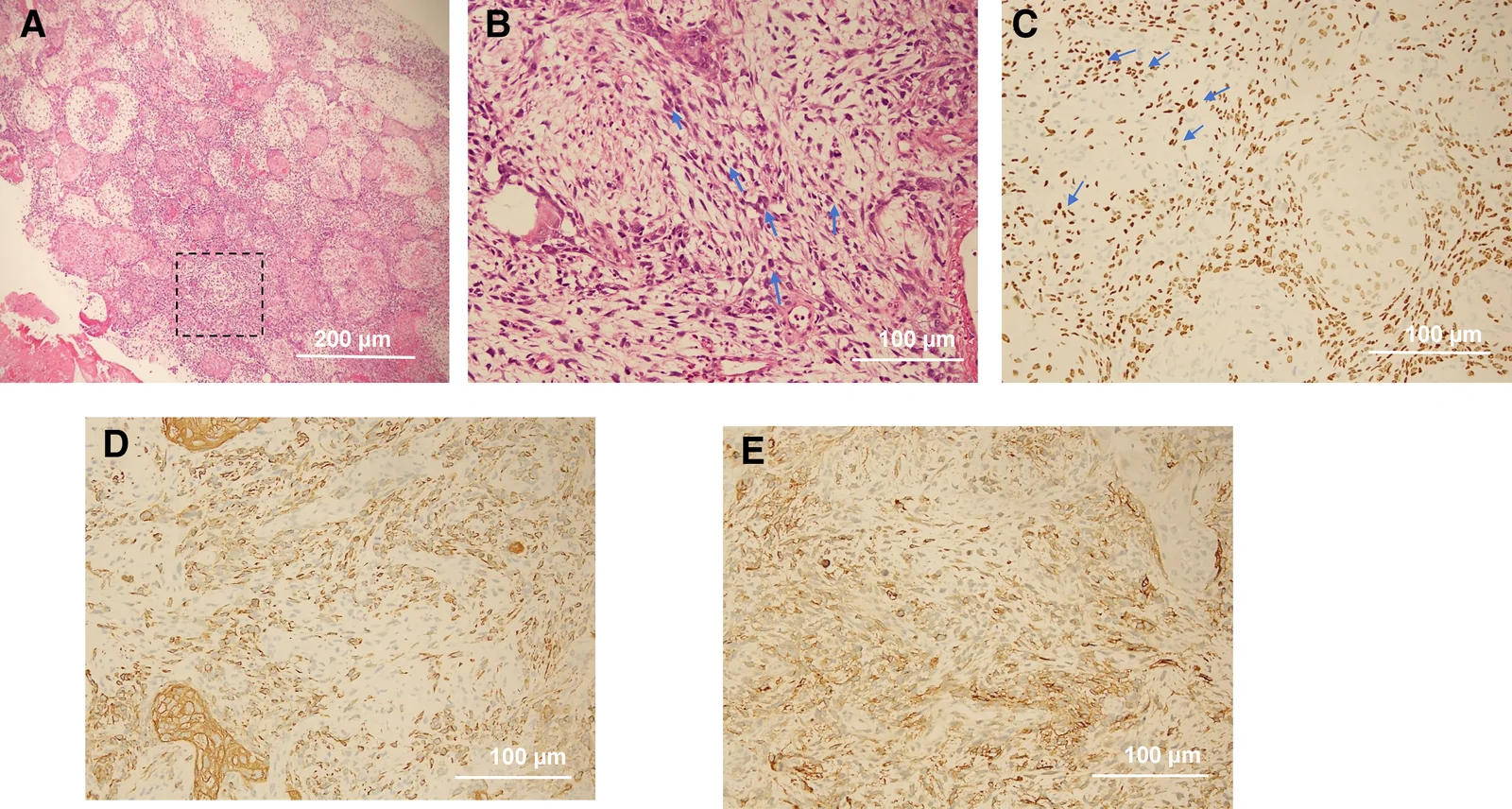
Histopathological and immunohistochemical features of the cervical biopsy specimen. (A) Low-magnification H&E staining (100×) reveals sheets of invasive squamous cell carcinoma; dashed boxes indicate the regions containing spindle-shaped cells; (B) high-power view (400×) of H&E-stained tissue shows spindle-shaped neoplastic cells with elongated, pleomorphic nuclei and identifiable mitotic figures (arrows); (C) immunohistochemical staining for p63 shows substantial nuclear staining throughout the tumor cell population (arrows); (D) immunohistochemical staining for CK shows diffuse cytoplasmic positivity in tumor cells, including the spindle cell component; (E) immunohistochemical staining for CD10 shows focal positive immunoreactivity in tumor cells. CK = cytokeratin, H&E = hematoxylin and eosin.

Serum squamous cell carcinoma antigen (SCC-Ag) was 18 ng/mL, and human papillomavirus-16 testing was positive. Pelvic magnetic resonance imaging (MRI) showed an irregular mass of 14 × 13 × 13 cm occupying most of the pelvic cavity (Fig. 1). Compression of both ureters had produced bilateral hydronephrosis. On gynecologic examination, a fused, gray-white, cauliflower-like lesion with a foul odor replaced the cervix and upper vagina and extended beyond half of the vaginal wall. Chest computed tomography (CT) and abdominal ultrasound found no distant metastases. Biopsies of the vagina and uterus showed squamous cell carcinoma with focal spindle cell features (Fig. 2). Immunohistochemistry was positive for p63 and cytokeratin, confirming the epithelial origin of the tumor cells, whereas cluster of differentiation 10 was only focally positive (Fig. 2). Histologic review together with the immunohistochemical profile excluded a second primary malignancy. The final diagnosis was FIGO stage IIIB (2021 criteria) CSCC with a partial spindle cell component.^[3]^

**Figure 3.**
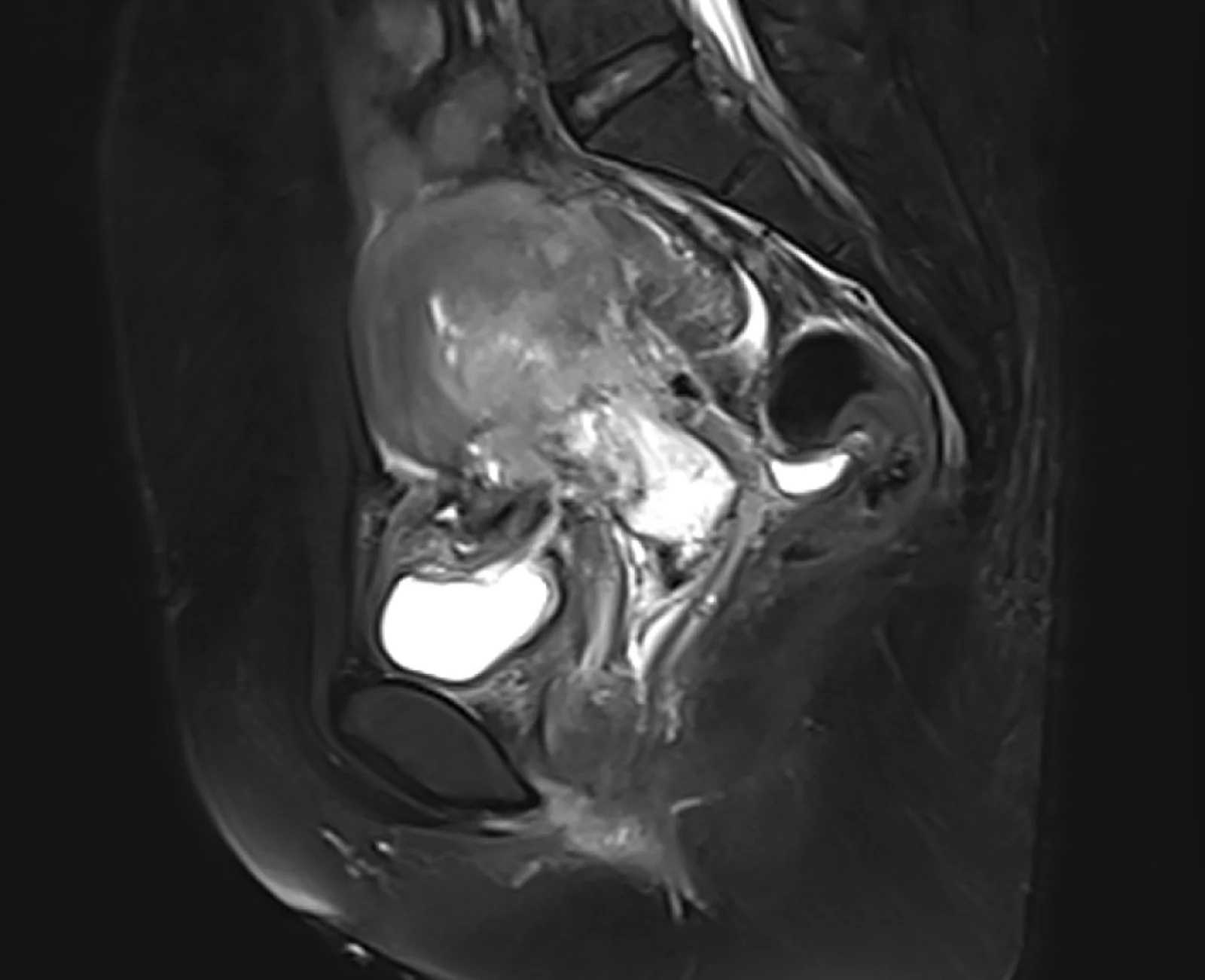
Post-NACT pelvic MRI. Sagittal T2-weighted fat-suppressed sequence obtained after 3 cycles of NACT (TP regimen) shows marked tumor shrinkage to 7.1 × 6.5 × 6.4 cm (arrows), corresponding to PR. The residual lesion remains hyperintense with irregular margins. MRI = magnetic resonance imaging, NACT = neoadjuvant chemotherapy, PR = partial remission, TP = paaclitaxel plus cisplatin.

From July to September 2023 she received 3 cycles of NACT with cisplatin 75 mg/m^2^ plus albumin-bound paclitaxel 260 mg/m^2^ at 21-day intervals. MRI after NACT showed that the mass had shrunk to 7.1 × 6.5 × 6.4 cm (Fig. 3), a partial remission, and SCC-Ag fell to 5.2 ng/mL. The regimen was tolerated well; grade 2 leukopenia was managed with granulocyte colony-stimulating factor. For personal reasons, treatment was then paused for 1 month.

**Figure 4.**
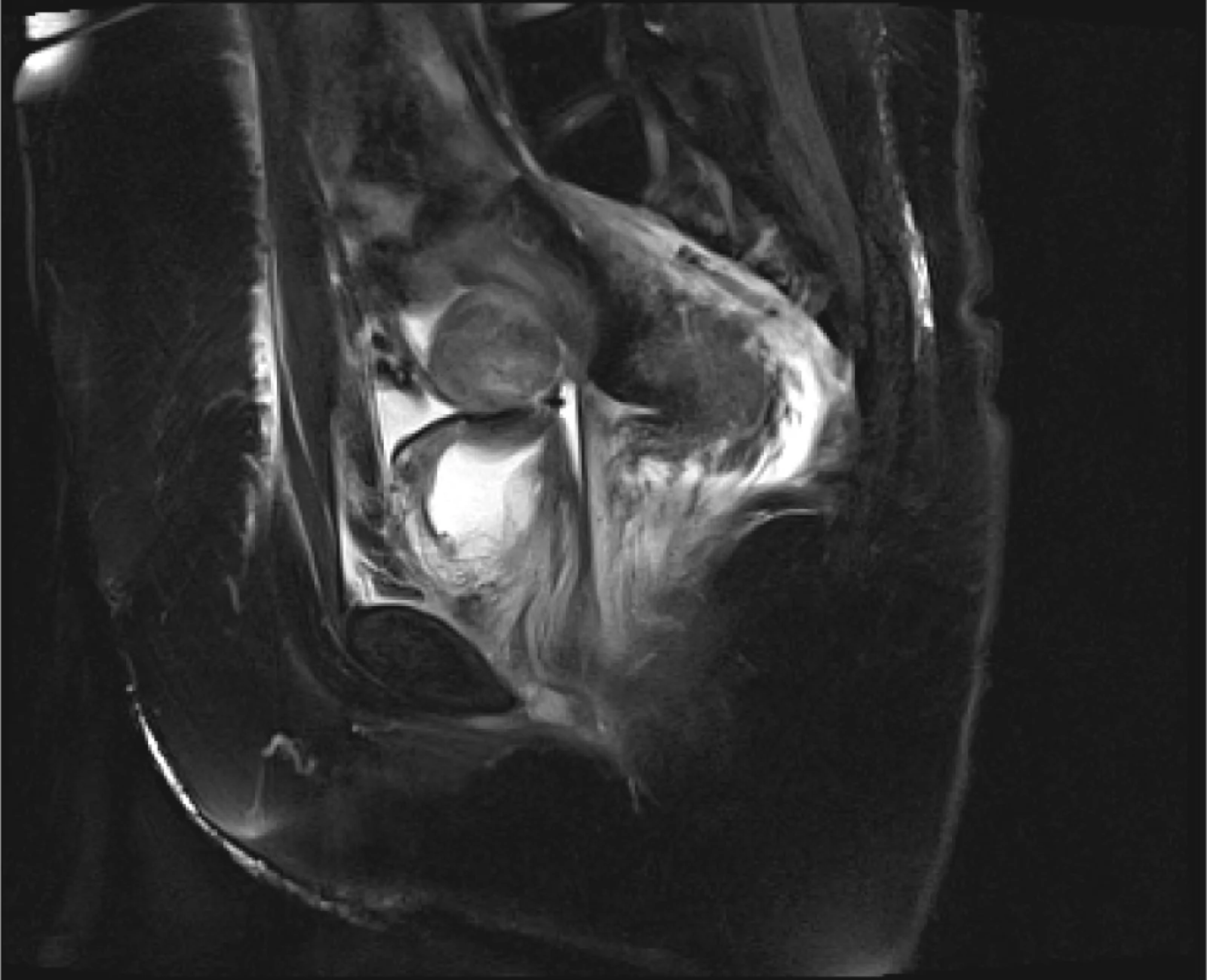
Posttreatment pelvic MRI. Sagittal T2-weighted fat-suppressed sequence acquired after CCRT and bevacizumab therapy demonstrates complete resolution of the previously visualized cervical mass, with no residual abnormal signal or soft tissue thickening, consistent with CR. CCRT = concurrent chemoradiotherapy, CR = complete remission, MRI = magnetic resonance imaging.

Positron emission tomography-CT in October 2023 detected enlarged lymph nodes beside the abdominal aorta and along both iliac vessels. From November 2023 to January 2024 the patient underwent volumetric modulated arc therapy with concurrent chemotherapy. The primary lesion received 70 Gy in 35 fractions, the enlarged lymph nodes 60 Gy, and the pelvic lymphatic drainage regions 50 Gy. During radiotherapy, 3 further cycles of the paaclitaxel plus cisplatin regimen at the same doses (21-day intervals) were given together with bevacizumab 800 mg every 3 weeks, which was continued as maintenance therapy for a total of 12 cycles. Bone marrow suppression and gastrointestinal reactions were grade 2 and manageable. Reevaluation in January 2024 showed CR on both clinical examination and MRI (Fig. 4), and SCC-Ag was below 1.5 ng/mL. The patient’s symptoms resolved completely after the full treatment course. Imaging at the 6-month follow-up (July 2024) showed no recurrence or metastasis. Because the patient relocated overseas, further follow-up was not possible.

## 3. Discussion

Three features of this case stand out: the exceptional size of the tumor (14 × 13 × 13 cm) with bilateral hydronephrosis from ureteral compression, the partial spindle cell component, and the complete response to sequential NACT, CCRT, and bevacizumab.

At this size, cervical tumors are usually not amenable to upfront surgery. Normal tissue planes are effaced and adjacent pelvic structures are invaded, so resection with negative margins is rarely achievable.^[3,4]^ In our patient, primary surgery would have carried a high risk of severe morbidity and incomplete resection. NACT was therefore used first, to reduce the tumor to a volume at which definitive CCRT could be delivered with acceptable toxicity. The paaclitaxel plus cisplatin regimen was chosen because platinum–taxane combinations have established activity in cervical cancer.^[8]^ After 3 cycles, the tumor volume had fallen by approximately 88% (calculated from the product of the 3 orthogonal diameters), indicating that massive CSCC can be highly chemosensitive. The biologic basis of this response is not fully understood, but the degree of shrinkage shows that platinum-based chemotherapy can be effective even in extremely bulky disease.

Positron emission tomography-CT after NACT detected nodal metastases at the para-aortic and iliac sites. Under the 2021 FIGO staging system, nodal involvement corresponds to stage IIIC, and para-aortic involvement specifically to IIIC2[3]. In this patient, the nodes were not evident at initial staging and were identified only after NACT, suggesting disease progression during the neoadjuvant window; micrometastases may grow while the primary lesion responds, a recognized risk of NACT-based strategies. The subsequent CCRT covered both the residual primary tumor and the involved nodes.

Bevacizumab was added to CCRT because of its anti-angiogenic action: it binds circulating VEGF, curbs the growth of tumor microvessels, and thereby limits the blood supply to the tumor.^[9]^ VEGF blockade has also been proposed to improve tumor perfusion and to enhance the efficacy of radiation and chemotherapy, although these effects are not fully characterized.^[8,10]^ Combined with pelvic radiotherapy, bevacizumab carries a theoretical risk of fistula formation; no such complication occurred in our patient. The complete morphologic response on MRI is consistent with additive or synergistic effects of this multimodal regimen.

The focal spindle cell morphology deserves attention for 2 reasons: it broadens the differential diagnosis and may carry prognostic implications. Sarcomatoid differentiation is uncommon in CSCC.^[11]^ When spindle cells are present, true sarcomas such as leiomyosarcoma and carcinosarcoma (malignant mixed Müllerian tumor) must be excluded before the lesion is classified as an epithelial tumor with spindle cell change.^[11,12]^ In our case, morphologic review and immunohistochemistry (Fig. 2) confirmed CSCC with focal spindle cell features and ruled out a sarcomatous component. The prognostic significance of spindle cell change in CSCC is uncertain; published data are limited, but some reports describe an aggressive clinical course in cervical sarcomatoid carcinoma.^[11,13]^ The favorable response in our patient suggests that CSCC with spindle cell features can still respond to platinum-based chemotherapy and radiation when treated aggressively.

The treatment sequence in this case, NACT followed by CCRT with bevacizumab, produced CR with short-term disease control. The rationale for this order is 2-fold. NACT rapidly debulks the primary tumor, so that CCRT can target a smaller and better-oxygenated volume, which is generally more radiosensitive; this is the classic argument for neoadjuvant approaches in bulky disease.^[4]^ At the same time, anti-angiogenic therapy may alter tumor vascularization and the microenvironment in ways that potentiate concurrent chemotherapy and radiotherapy.^[8,10]^ These observations are in line with earlier reports of sequenced multimodal therapy in bulky gynecologic malignancies.

This work has clear limitations. It is a single retrospective case, so the findings cannot be generalized and may be subject to selection and reporting bias; similar cases with unsuccessful outcomes may simply have gone unreported. Six months of follow-up is too short to assess long-term disease-free survival and late toxicities. In addition, the patient declined brachytherapy after achieving CR, so the primary lesion received only external beam radiotherapy (70 Gy in 35 fractions; equivalent dose in 2-Gy fractions = 70 Gy, *α*/*β* = 10), below the ≥ 85 to 90 Gy equivalent dose in 2-Gy fractions recommended by the Experience‑Based Multicenter Review of Cervical Cancer consortium for definitive treatment of locally advanced cervical cancer^[14]^; the risk of local recurrence is therefore a concern. Larger prospective studies are needed to confirm the efficacy and safety of this combined approach.

## 4. Conclusion

A stage IIIB CSCC presenting as a 14 × 13 × 13 cm mass with a spindle cell component achieved CR after a carefully sequenced regimen of NACT, CCRT, and bevacizumab. The case supports an individualized, stepwise multimodal approach to bulky cervical tumors. Whether this strategy can be applied more broadly must be tested in prospective studies with longer follow-up.

## Author contributions

**Conceptualization:** Hua Jin.

**Investigation:** Hua Jin.

**Methodology:** Hua Jin.

**Resources:** Hua Jin.

**Validation:** Hua Jin.

**Writing – original draft:** Hua Jin, Weibo Wen.

**Writing – review & editing:** Hua Jin.

**Funding acquisition:** Zhengri Piao.

**Supervision:** Zhengri Piao.

## References

[1] Wu J Jin Q Zhang Y. Global burden of cervical cancer: current estimates, temporal trend and future projections based on the GLOBOCAN 2022. J Natl Cancer Cent. 2025;5:322–9. 40693230 10.1016/j.jncc.2024.11.006 PMC12276544

[2] Zheng S Li R Liang J. Serum miR-638 combined with squamous cell carcinoma-related antigen as potential screening biomarkers for cervical squamous cell carcinoma. Genet Test Mol Biomarkers. 2020;24:188–94. 32216635 10.1089/gtmb.2019.0147

[3] Bhatla N Aoki D Sharma DN Sankaranarayanan R. Cancer of the cervix uteri: 2021 update. Int J Gynaecol Obstet. 2021;155(Suppl 1):28–44. 34669203 10.1002/ijgo.13865 PMC9298213

[4] Kenter GG Greggi S Vergote I. Randomized phase III study comparing neoadjuvant chemotherapy followed by surgery versus chemoradiation in stage IB2–IIB cervical cancer: EORTC 55994. J Clin Oncol. 2023;41:5035–43. 37656948 10.1200/JCO.22.02852

[5] Rose PG Bundy BN Watkins EB. Concurrent cisplatin-based radiotherapy and chemotherapy for locally advanced cervical cancer. N Engl J Med. 1999;340:1144–53. 10202165 10.1056/NEJM199904153401502

[6] Zhao FJ Su Q Zhang W Yang WC Zhao L Gao LY. Endu combined with concurrent chemotherapy and radiotherapy for stage IIB-IVA cervical squamous cell carcinoma patients. World J Clin Cases. 2021;9:8061–70. 34621863 10.12998/wjcc.v9.i27.8061 PMC8462205

[7] Yang SL Chen L He Y Zhao H Wu YM. Effect of neoadjuvant chemotherapy followed by surgery for FIGO stage I-II cervical cancer: a meta-analysis. J Int Med Res. 2020;48:300060520945507. 32867558 10.1177/0300060520945507 PMC7469733

[8] Tewari KS Sill MW Long HJ 3rd. Improved survival with bevacizumab in advanced cervical cancer. N Engl J Med. 2014;370:734–43. 24552320 10.1056/NEJMoa1309748 PMC4010094

[9] Kazazi-Hyseni F Beijnen JH Schellens JH. Bevacizumab. Oncologist. 2010;15:819–25. 20688807 10.1634/theoncologist.2009-0317 PMC3228024

[10] Watkins DE Craig DJ Vellani SD. Advances in targeted therapy for the treatment of cervical cancer. J Clin Med. 2023;12:5992. 37762931 10.3390/jcm12185992 PMC10531664

[11] Brown J Broaddus R Koeller M Burke TW Gershenson DM Bodurka DC. Sarcomatoid carcinoma of the cervix. Gynecol Oncol. 2003;90:23–8. 12821337 10.1016/s0090-8258(03)00200-2

[12] Hrudka J Rosová B Halaška MJ. Squamous cell carcinoma with sarcomatoid differentiation or carcinosarcoma of the uterine cervix associated with HPV33 infection: a case report. Diagn Pathol. 2020;15:12. 32035484 10.1186/s13000-020-00934-y PMC7007670

[13] Mohan H Garg S Handa U. Sarcomatoid carcinoma of the cervix: case report of a rare entity. Arch Gynecol Obstet. 2008;277:571–3. 18034352 10.1007/s00404-007-0512-4

[14] Pötter R Tanderup K Kirisits C. The EMBRACE II study: the outcome and prospect of two decades of evolution within the GEC-ESTRO GYN working group and the EMBRACE studies. Clin Transl Radiat Oncol. 2018;9:48–60. 29594251 10.1016/j.ctro.2018.01.001 PMC5862686

